# Wide-Awake Hemi-hamate Arthroplasty for Chronic PIPJ Fracture-Dislocation: A Case Report

**DOI:** 10.5704/MOJ.2311.014

**Published:** 2023-11

**Authors:** IJ Magtoto

**Affiliations:** Hand and Reconstructive Microsurgery Unit, Philippine Orthopedic Center, Quezon City, Philippines

**Keywords:** WALANT, PIPJ fracture-dislocation, hemi-hamate arthroplasty

## Abstract

Wide-awake local anaesthesia, no tourniquet (WALANT) has been reported for upper extremity procedures of varying complexities ranging from simple tendon repairs to more complicated soft tissue and bony reconstructions. Hemi-hamate arthroplasty under WALANT has yet to be described in English literature. We report a case of a chronic dorsal PIPJ fracture-dislocation who underwent open reduction followed by Hemi-hamate Arthroplasty under wide-awake anaesthesia. There was adequate visualization during the surgery with no additional anaesthesia required. Active intra-operative range of motion and joint stability testing was possible with no pain experienced throughout the procedure. 10-month post-operative follow-up showed excellent range of motion with occasional tolerable pain during maximal finger flexion and power grip. Wide-awake anaesthesia is a viable and safe alternative for hemi-hamate arthroplasty.

## Introduction

Unstable Proximal Interphalangeal Joint (PIPJ) fracture-dislocations are common injuries that pose a unique and complex challenge for the treating hand surgeon^1,2^. Chronic injuries are more often unpredictable and various treatment options combined with open reduction such as bone grafting, volar plate arthroplasty, external fixation, and hemi-hamate arthroplasty have been described^2^. Wide-awake, local anaesthesia, no tourniquet (WALANT) was popularised by Lalonde^3^ and has been gaining increasing popularity especially during the time of the COVID pandemic^4^. Hemihamate arthroplasty using wide-awake anaesthesia has not yet been reported in English literature.

We present a patient who presented with a chronic PIPJ fracture-dislocation who underwent open reduction and hemi-hamate arthroplasty under wide-awake anaesthesia.

## Case Report

A 27-year-old male, right-handed office worker came to our clinic for persistent pain and limitation of range of motion of their right middle finger after sustaining an injury during a basketball game 4 months prior. No previous consult was sought.

**Fig 1:**
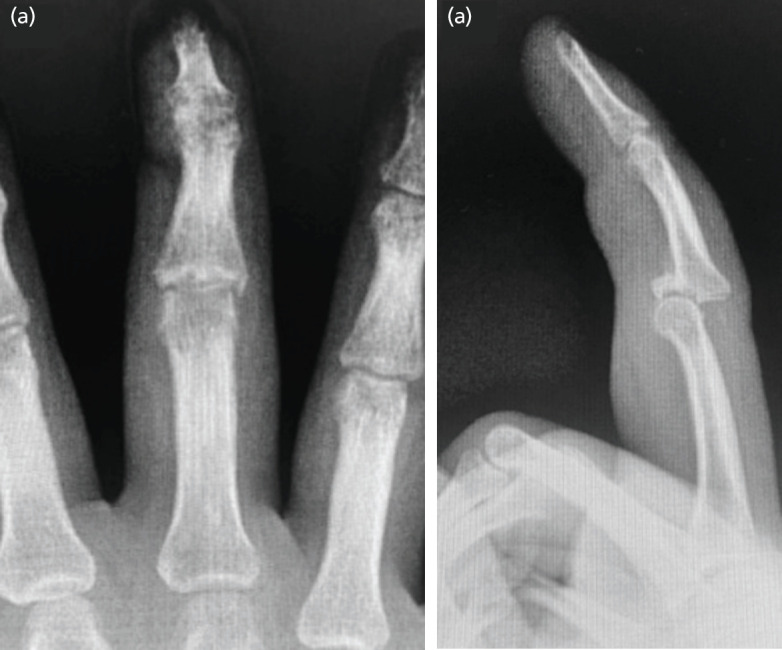
(a) Middle finger AP and (b) lateral radiographs demonstrating a comminuted volar lip fracture involving approximately 40% of the articular surface of the middle phalanx with dorsal dislocation of the PIPJ.

On physical examination, the patient’s right middle finger PIPJ was noted to be slightly swollen with limited arc of motion from 0° to 20°. No neurovascular compromise was noted. Radiographs of the patient’s middle finger revealed a comminuted dorsal PIPJ fracture-dislocation involving approximately 40% of the articular surface (Fig. 1). An open reduction with hemi-hamate arthroplasty under wide-awake anaesthesia was suggested for the patient.

**Fig 2:**
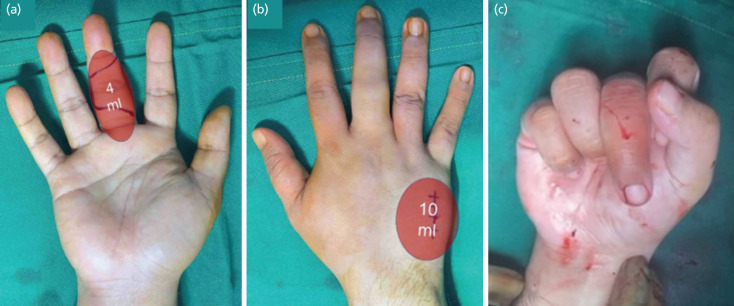
Intra-operative images. (a , b) Planned operative incisions with location of local anaesthetic infiltration and corresponding volumes. (c) Intra-operative range of motion testing done after fixation of the graft demonstrating a stable joint with PIPJ flexion up to 90°.

The anaesthetic solution used was a buffered 1% lidocaine with 1:100,000 epinephrine. An ultrasound-assisted median and ulnar nerve block was performed by the surgeon using a wireless linear ultrasound probe (L15 HD2, Clarius, Canada, frequency 5–15 MHz) and was done at the mid forearm using 3ml of anaesthetic solution for each nerve. These nerve blocks are optional and may be omitted depending on the surgeon’s preference. Ten (10) ml of the same solution was then used to infiltrate the subcutaneous area and periosteum of the 4th and 5th carpometacarpal joint (CMCJ) followed by an additional 4ml for the volar surface of the middle finger (Fig. 2a, b). A total of 20ml of solution was used.

A modified Bruner incision was utilized and a shot-gun approach to the PIPJ was done^1^. The size of the defect of the volar lip of the middle phalangeal base was measured which determined the graft size for the hamate. A longitudinal incision was then done over the dorsal wrist centred over the 4th and 5th CMCJ. The graft was harvested using an oscillating saw and osteotome, trimmed and then fixed using two 1.5mm screws. Proper graft position, implant placement and joint reduction was confirmed with intra-operative radiographs.

**Fig 3:**
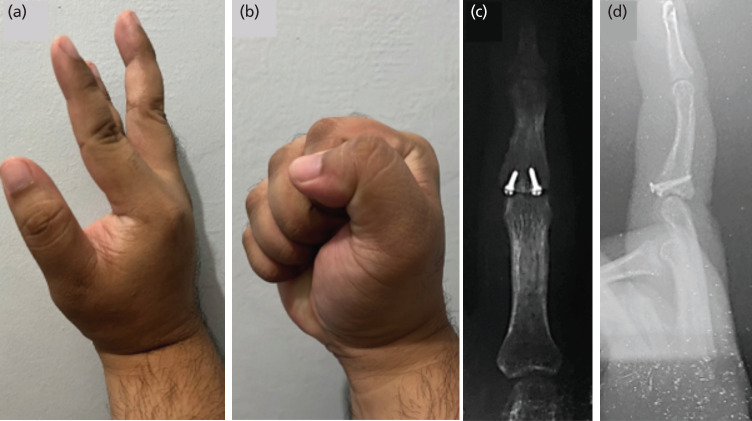
(a) 10-month post-operative outcome of finger range of motion demonstrating full PIPJ extension, and (b) flexion up to 90° and (c, d) post-operative radiographs showing bony union and concentric PIPJ joint reduction.

Wide-awake anaesthesia allowed for active intra-operative range of motion and stability testing (Fig. 2c). The patient’s Pain Numerical Rating Scale (NRS) throughout the operation was zero and was discharged the following day with oral, non-opiate analgesics. His finger was initially immobilised in a finger splint with his PIPJ in 30° of flexion. On his 5th post-operative day, he was placed on a dorsal blocking splint which limited PIPJ extension to 30° with no limitation of flexion. The splint was discontinued at six weeks and was allowed for light-use of his hand. At 10 months post-operative follow-up, the patient had near-full flexion of his middle finger with radiographs demonstrating union and joint congruity (Fig. 3). The patient was able to return to his work with no difficulty typing using the computer, however, reported occasional tolerable pain at the dorsal area of his PIPJ at 1-2 NRS during maximal flexion and power grip. At 18 months post-operative follow-up via phone interview, the patient was able to perform light boxing at his gym.

## Discussion

Since its introduction by Lalonde, WALANT has been increasingly popular and has been described in various hand and extremity procedures^3,4^. It is also the preferred option during the COVID pandemic since it avoids aerosol-generating procedures such as intubation, reduces needed manpower since an anaesthesiologist is not required, provides adequate pain control, and allows for rapid patient turn-over^4^.

Hemi-hamate arthroplasty has been reported to have the most consistent outcome for treatment of chronic dorsal PIPJ fracture-dislocations, especially if there is more than 50% joint involvement and comminuted fracture fragments^1,2^. For our patient, we were able to achieve excellent results with near full finger range of motion. However, there was still mild pain during full flexion and power grip, which is also consistent with the previously reported results and could be possibly explained by arthritic changes due to the chronicity of the injury^1,2,5^. Wide-awake anaesthesia also allowed adequate visualisation of the operative field without the need for a tourniquet. This avoids the necessity of performing sedation or proximal nerve blocks to address tourniquet pain^3,4^.

Advantages of doing the procedure using wide-awake anaesthesia would include obviating the need for preoperative laboratory testing and fasting and reducing overall cost. Although we did this procedure as an in-patient procedure, this could have very well been done in an outpatient setting since WALANT reduces the need for postoperative anaesthesia monitoring, allowing for immediate discharge after the procedure^4^. Pain control throughout the procedure was satisfactory and there was no need to take opiate analgesics. It allows for dynamic intra-operative stability and range of motion testing after placement of the graft, although this remains to be seen if it has any long-term clinical benefit as most of the previously reported chronic PIPJ reconstructions did not experience any dislocations despite using the conventional anaesthesia^1,2,5^. Also, even though this was done in a chronic injury, this approach is also feasible to use in acute cases. Contraindications to WALANT would include allergy to lidocaine or epinephrine, uncooperative patients, and patient refusal.

Hemi-hamate arthroplasty under wide-awake anaesthesia has not been previously described and is a viable, simple, and safe alternative to conventional regional or general anaesthesia. Considering all the benefits of WALANT, we would recommend the use of wide-awake anaesthesia as the primary anaesthetic technique of choice, unless contraindicated, for hemi-hamate arthroplasty.
